# Longitudinal *In Vivo* Imaging of Retinal Ganglion Cells and Retinal Thickness Changes Following Optic Nerve Injury in Mice

**DOI:** 10.1371/journal.pone.0040352

**Published:** 2012-06-29

**Authors:** Balwantray C. Chauhan, Kelly T. Stevens, Julie M. Levesque, Andrea C. Nuschke, Glen P. Sharpe, Neil O'Leary, Michele L. Archibald, Xu Wang

**Affiliations:** 1 Retina and Optic Nerve Research Laboratory, Dalhousie University, Halifax, Nova Scotia, Canada; 2 Department of Physiology and Biophysics, Dalhousie University, Halifax, Nova Scotia, Canada; 3 Department of Ophthalmology and Visual Sciences, Dalhousie University, Halifax, Nova Scotia, Canada; Institute Biomedical Research August Pi Sunyer (IDIBAPS) – Hospital Clinic of Barcelona, Spain

## Abstract

**Background:**

Retinal ganglion cells (RGCs) die in sight-threatening eye diseases. Imaging RGCs in humans is not currently possible and proof of principle in experimental models is fundamental for future development. Our objective was to quantify RGC density and retinal thickness following optic nerve transection in transgenic mice expressing cyan fluorescent protein (CFP) under control of the Thy1 promoter, expressed by RGCs and other neurons.

**Methodology/Principal Findings:**

A modified confocal scanning laser ophthalmoscopy (CSLO)/spectral-domain optical coherence tomography (SD-OCT) camera was used to image and quantify CFP+ cells in mice from the B6.Cg-Tg(Thy1-CFP)23Jrs/J line. SD-OCT circle (1 B-scan), raster (37 B-scans) and radial (24 B-scans) scans of the retina were also obtained. CSLO was performed at baseline (n = 11) and 3 (n = 11), 5 (n = 4), 7 (n = 10), 10 (n = 6), 14 (n = 7) and 21 (n = 5) days post-transection, while SD-OCT was performed at baseline and 7, 14 and 35 days (n = 9) post-transection. Longitudinal change in CFP+ cell density and retinal thickness were computed. Compared to baseline, the mean (SD) percentage CFP+ cells remaining at 3, 5, 7, 10, 14 and 21 days post-transection was 86 (9)%, 63 (11)%, 45 (11)%, 31 (9)%, 20 (9)% and 8 (4)%, respectively. Compared to baseline, the mean (SD) retinal thickness at 7 days post-transection was 97 (3)%, 98 (2)% and 97 (4)% for the circle, raster and radial scans, respectively. The corresponding figures at 14 and 35 days post-transection were 96 (3)%, 97 (2)% and 95 (3)%; and 93 (3)%, 94 (3)% and 92 (3)%.

**Conclusions/Significance:**

Longitudinal imaging showed an exponential decline in CFP+ cell density and a small (≤8%) reduction in SD-OCT measured retinal thickness post-transection. SD-OCT is a promising tool for detecting structural changes in experimental optic neuropathy. These results represent an important step towards translation for clinical use.

## Introduction

The eye provides a unique opportunity to image central nervous system tissue *in vivo* because of the transparent cornea and crystalline lens allowing direct optical visualization of the retina. The retina is laminated and highly organized neural tissue studied widely in neuroscience. [1] It is affected in the many visually disabling and blinding diseases. Macular degeneration causes damage to the outer retina, including the rod and cone photoreceptors, and the retinal pigment epithelium in the outer retina, [2] while glaucoma and other optic neuropathies cause damage to the retinal ganglion cells (RGCs), situated in the inner retina. [3] RGC axons form the optic nerve and in primates project primarily to targets in the lateral geniculate nuclei, but also others such as the superior colliculi and pretectal nuclei. In rodents, RGC axons synapse primarily in the superior colliculi, but project also to other minor targets.

Because of their clinical significance, studies of optic neuropathies under experimental conditions are vital for understanding their pathophysiology and devising potential therapeutic strategies. Methods for estimating RGC loss after experimental damage, or RGC survival in neuroprotective studies, in rodents have relied almost exclusively on quantifying either retrogradely labeled RGC soma after application of a fluorescent tracer to the superior colliculus, [4], [5] or axons in optic nerve sections. [6], [7] Since introduction of the fluorescent tracer to the superior colliculus is invasive, potential retrograde damage may occur to RGCs confounding the results of the primary experiment. Additionally, since with this method quantification of RGC survival requires isolating the retina, each animal can provide only one time point and any longitudinal assessment assumes that the actual time-course in an individual animal can be extrapolated from data points contributed cross-sectionally by multiple animals. This assumption is likely tenuous and could potentially lead to inaccurate results, while inter-animal variability in the number of RGCs and individual susceptibility to injury increases the number of animals required for statistical testing. The ability to image the same animal longitudinally over a period of time to quantify RGC survival is therefore a desirable attribute in studies of RGC damage.

The availability of transgenic mouse lines in which fluorescent proteins are expressed under the control of an RGC protein has accelerated the progress of research in the longitudinal rate of RGC decline after injury. In the Thy1-CFP line, cyan fluorescent protein (CFP) is expressed under the modified Thy1 gene promoter. [8] Thy1 expression occurs in around 80% of RGCs [8], [9] and is thought to be strong and stable. [8] With a modified confocal scanning laser ophthalmoscope (C), *in vivo* imaging has been described in the conscious Thy1-CFP mouse. [10]

Recent developments in spectral domain optical coherence tomography (SD-OCT) permits unprecedented image quality in human retinal imaging, both in *en face* and cross-section (B-scan) [11] modes. The ability of these systems to acquire images adjusting for eye movements in real-time has significant advantages in clinical practice. [12] Since eye and head movements are also factors for loss of image quality in anesthetized animals because of respiration, real-time correction likely also offers significant benefits in animal imaging. Furthermore, image registration allows imaging to occur in the identical locations over time such that an accurate assessment of RGC loss can be made.

The purpose of this study was to exploit the advantages offered by image registration and increased signal-noise ratio in CSLO and SD-OCT to quantify the decline of the RGC population and changes in retinal thickness after optic nerve transection (ONT).

## Methods

### Ethics statement

All procedures complied with the standards of the Canadian Council of Animal Care and ethics approval was obtained from the Dalhousie University Committee on Laboratory Animals (Protocol number: 10–011).

### Animals

Adult Thy1-CFP transgenic (Strain: B6.Cg-Tg(Thy1-CFP)23Jrs/J) and wild type mice were purchased from the Jackson Laboratory (Bar Harbor, ME) as breeders. Mice were housed and bred in a 12-hour light-dark cycle environment and given food and water *ad libitum*. Mice were genotyped to confirm the inclusion of the Thy1-CFP transgene as described previously. [9] This strain of mouse was chosen as it has been used most frequently in this line of research.

A total of 30 animals (aged 16–20 weeks) in three groups were used: group 1 (n = 13), for quantifying CFP positive (CFP+) cells after ONT; group 2 (n = 13), for quantifying change in retinal thickness after ONT with SD-OCT and group 3 (n = 4), for quantifying CFP levels during anesthesia.

### *In vivo* imaging technique

A modified CSLO/SD-OCT camera (Spectralis, Heidelberg Engineering GmbH, Heidelberg, Germany) was used in these experiments to image the central retina. In CSLO mode, the system permits fundoscopic imaging under infrared (820 nm) illumination. Acquisition software allows real-time retinal tracking such that the same location is imaged during an imaging session eliminating motion artifacts due, for example, to respiration. In this manner, multiple images can be acquired in the same locked location increasing the signal-noise ratio. The software also locks the same location in subsequent imaging sessions so that the identical area can be imaged longitudinally.

A special excitation bandpass (448 nm) and barrier (460–490 nm) filter set were introduced into the camera to permit imaging of CFP+ cells. Images subtending 30°, centred on the optic nerve head, were acquired and averaged (∼ 20 *en face* CSLO images). At baseline, care was taken to optimally focus the image at the level of the CFP+ cells. In the follow-up images after ONT, the same transverse image plane as baseline was locked in. The same focus settings as baseline were also used and adjusted in small steps (± 0.5 D) to obtain images in the optimal plane. Additionally, *en face* infrared images of the retinal nerve fibre layer (RNFL) consisting of bundles of RGC axons were obtained with infrared CSLO.

**Figure 1:**
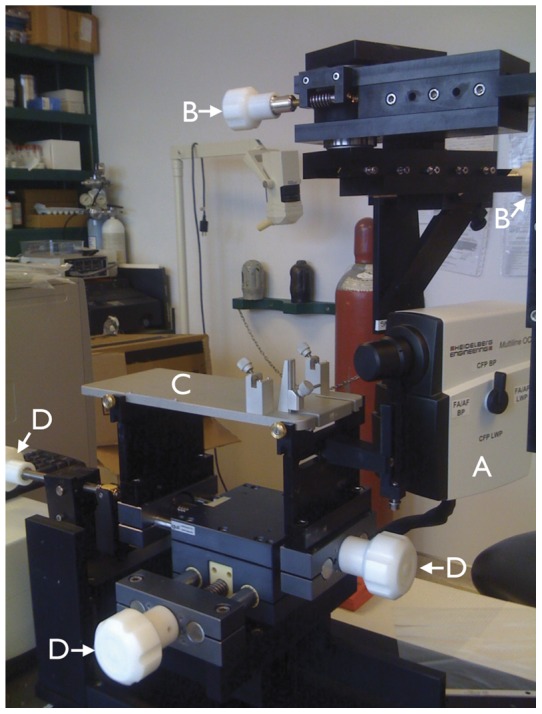
Scanning laser ophthalmoscopy/spectral domain optical coherence tomography set up. Camera (A) mounted on a customized stereotaxic frame which allowed rotation along the horizontal and vertical axes with two geared systems (controlled by knobs B). The top plate of a rodent stereotaxic frame (C; the ear and bite bar holders were removed for these experiments) was positioned along the horizontal and vertical axes (controlled by knobs D).

The CSLO/SD-OCT camera was mounted on a customized stereotaxic frame allowing it to be rotated in the horizontal and vertical planes around a centre-point. The top plate of a rodent stereotaxic frame (Model 51400, Stoelting, Wood Dale, IL) was mounted on a base plate which could be moved laterally and vertically (Fig. 1).

**Figure 2:**
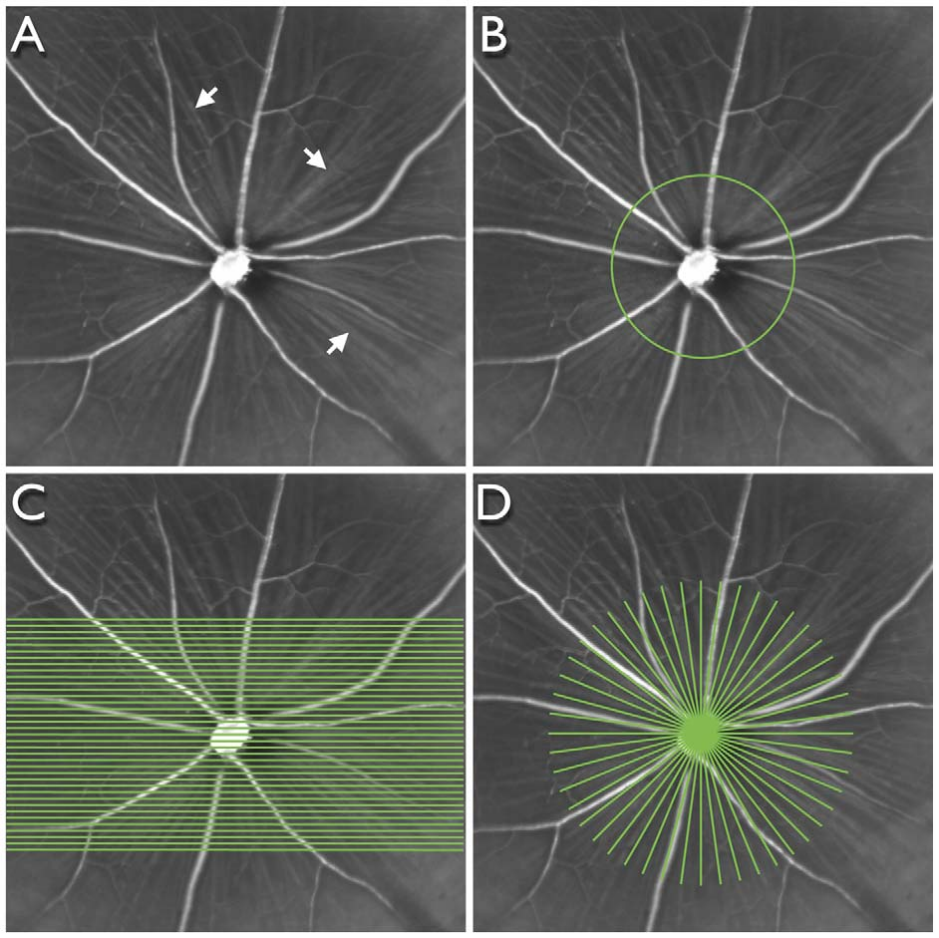
Scanning patterns used for spectral domain optical coherence tomography. Infrared image of a mouse retina (A) centred on the optic nerve head showing the retinal blood vessels and retinal nerve fibre layer (arrows). Scanning patterns centred on the optic nerve head: circular (B), raster (C) and radial (D).

The principles of SD-OCT have been described elsewhere, [13] however briefly, it is a non-invasive technology using low-coherence interferometry to generate high-resolution cross-sectional images of ocular structures. The reference beam and reflected beam from the eye are recorded in parallel by a spectrometer to generate A-scans from Fourier-transformed time-delayed signals. Each SD-OCT session comprised imaging with 3 scan patterns centred on the optic nerve head (Fig. 2): (1) a circular B-scan subtending 12°; (2) a raster pattern of 37 equally spaced horizontal B-scans, each subtending 30° and (3) a radial pattern of 24 angularly equidistant B-scans, each subtending 20°. The scanning speed was 40,000 A-scans per second and each B-scan comprised either 1536 (circle and raster) or 1024 (radial) A-scans. Each B-scan was averaged 16 times.

### *In vivo* imaging protocol

Before imaging, pupils were dilated with a topical mydriatic (0.8% tropicamide and 5% phenylephrine hydrochloride, Sandoz Canada Inc., Boucherville, QC). Animals were anesthetized with isofluorane (Baxter Corporation, Mississauga, ON) with a portable isofluorane inhalation system (Summit Medical Equipment Company, Bend, OR) after initial induction in a chamber with 2% (vol) isofluorane at 2 L/min oxygen flow. Once the mouse was lightly anesthetized it was transferred to the sterotaxic frame with a heating pad and rectal probe to maintain body temperature. For CSLO imaging, a plano (0 D) polymethyl methacrylate contact lens (Cantor and Nissel, Brackley, UK) was placed on the cornea. For SD-OCT, a concave (−30 D) contact lens (Contact Lens Laboratory, Queen Elizabeth Health Sciences Centre, Halifax, Canada) was used. The diameter of both lenses was 3.2 mm. Anesthesia was maintained by applying 1.5–1.8% isofluorane via a nose cone at 0.8 L/min oxygen flow throughout the imaging session. After imaging, the isofluorane vaporizer was shut off; the oxygen flow was maintained until the mouse awoke from anesthesia, usually within 1 min.

Baseline CSLO or SD-OCT imaging was performed on the left eye of each animal. Group 1 animals were reimaged periodically after ONT and immediately prior to sacrifice at either 3, 7, 14 or 21 days. Group 2 animals were re-imaged at 7, 14 and 35 days after ONT and sacrificed immediately after imaging at 35 days (n = 8). Control animals (n = 4) were imaged at 0, 3 and 7 days with all 3 scanning patterns to determine the total and within-session variability of SD-OCT measurements. These animals did not undergo ONT. One animal was sacrificed immediately after baseline imaging, without ONT, for comparing SD-OCT with histology. A summary of the imaging schedule and sample sizes is shown in Table 1.

**Table 1:** Imaging schedule and sample size at each time point.

	Day sacrificed	Day							
		BL (0)	3	5	7	10	14	21	35
CFP imaging									
	3	1	1						
	7	3	3	1	3				
	14	2	2	2	2	2	2		
	21	5	5	1	5	4	5	5	
SD-OCT imaging									
	0	1							
	35				8		8		8
		4*	4*		4*				

CFP, cyan fluorescent protein.

SD-OCT, spectral domain optical coherence tomography.

BL, baseline (prior to optic nerve transection).

All observations after BL are post optic nerve transection, except *which represent control (non optic nerve transected) animals for estimating SD-OCT variability.

Either CSLO or SD-OCT imaging was completed within 20 mins. To maintain optimal animal condition and not compromise image quality, either CSLO (Group 1) or SD-OCT (Group 2) was performed to reduce imaging time. To investigate whether isofluorane anesthesia affected the expression of the Thy1 promotor, hence potentially influencing quantification of CFP+ cells, another group of 4 mice (group 3) were used to quantitate retinal CFP expression after 0 (n = 2; 4 eyes) and 30 (n = 2; 4 eyes) mins of isofluorane anesthesia.

### Optic nerve transection

Under anesthesia, as described above, the globe was rotated downwards with a 9–0 conjunctival suture. An incision was made in the skin near the supraorbital ridge after which the intraorbital subcutaneous tissues were dissected to expose the optic nerve. The optic nerve dura was cut longitudinally along the superior aspect and the optic nerve carefully and completely transected approximately 0.5 mm from the globe. Care was taken to ensure there was no damage to the ophthalmic artery located underneath the transected nerve. After the incision was closed, the fundus was examined with the operating microscope to confirm no ischemic damage.

### Tissue preparation

All animals were sacrificed with an overdose of sodium pentobarbital (100 mg/kg bodyweight) by intraperitoneal injection. For one animal in group 2, the eyes were transferred to 30% sucrose overnight, horizontally sectioned at 20 µm thickness, mounted on slides and processed for cresyl violet staining. For group 3, retinas were isolated, flash frozen in liquid nitrogen and stored at –80°C.

### Western blot analysis

Western blot analysis was used to quantify CFP expression in the retina in Group 3 animals. Proteins from each retina were isolated individually in 50 µl of radioimmunoprecipitation assay buffer supplemented with protease inhibitors cocktail (Sigma, St. Louis, MO, USA). The protein extracts (5 μg of protein) were run on 10% SDS-polyacrylamide gels and transferred to immobilon P transfer membrane (Millipore Corporation, Bedford, MA). Membranes were then blocked in 5% fat free dry milk and 0.1% Tween 20 in Tris-buffered saline, cut into 2 pieces and incubated overnight with rabbit anti-CFP (1∶200; US Biological, Swampscott, MA) and rabbit polyclonal anti-actin (1∶1000; Sigma, St. Louis, MO) separately at 4°C. The immunoreactive bands were visualized with horseradish peroxidase conjugated anti-rabbit antibody (1∶1000; Vector Labs, Burlington, ON) and the Western-blotting detection system (ESL, Amersham Biosciences Piscataway, NJ). Films were scanned and band optical density measured with image analysis software (ImageJ, National Institutes of Health, Bethesda, MD). Protein levels were normalized using the anti-actin data for each sample.

### Data analysis

The *in vivo* CSLO images showing the CFP+ cells were exported to image analysis software (ImageJ). Cells were manually counted using the “Cell Counter” plug-in. The experimenter was masked to the animal code and time point (baseline and post-ONT). In longitudinal *in vivo* images, CFP+ cells were counted in the same area of the retina and density measurements derived. The change in the density of CFP+ cells was computed as a proportion of the baseline (pre-ONT) value.

Total retinal thickness was measured from the vireo-retinal surface to the retinal pigment epithelium/Bruch's membrane complex as delineated by the segmentation algorithm (Heidelberg Eye Explorer, Heidelberg Engineering). The outer border of the retinal pigment epithelium/Bruch's membrane complex was delineated as this layer is relatively thick in rodents. Each averaged B-scan was checked for segmentation failures and manually corrected.

The change in CFP+ cell density and retinal thickness after ONT was measured with a repeated measures analysis of variance. Model fitting with candidate fits was tested with a F-test to determine the best fit. Descriptive statistics were reported with parametric methods. Group comparisons were made with a t-test. All statistical tests were two-tailed and significance was assumed when *P*<0.05.

## Results

The mean (SD) CFP level in retinas of animals exposed to 30 mins of isofluorane was non-significantly elevated by 13 (11)% compared to animals not exposed to isofluorane (*P* = 0.19). The effects of isofluorane anesthesia are therefore unlikely to have had an effect on CFP expression and quantification of CFP+ cells.

**Figure 3:**
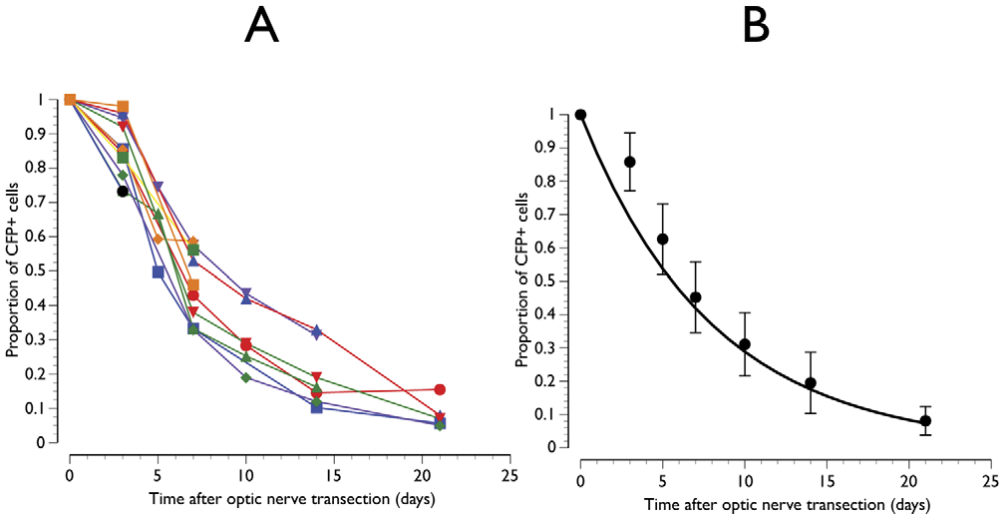
Proportion of cyan fluorescent protein positive (CFP+) cells surviving after optic nerve transection. Individual animal (A) and mean data with line showing the exponential function fitted though x = 0 and y = 1 (B). Error bars represent standard deviations.

**Figure 4:**
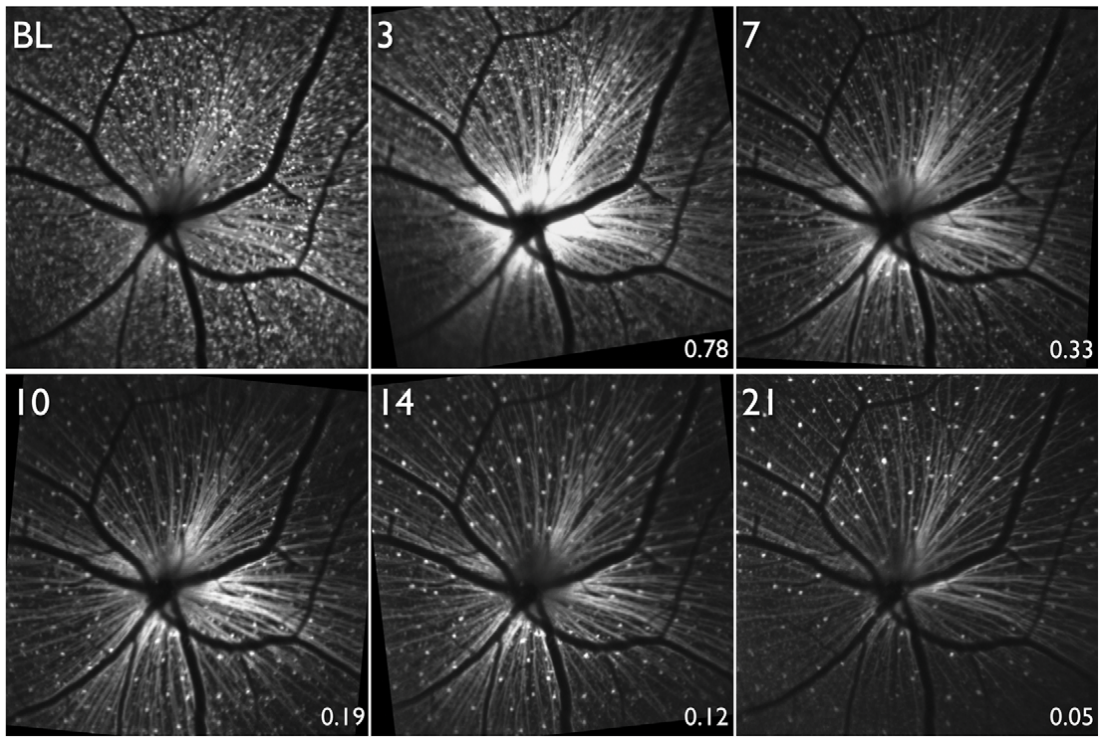
Longitudinal scanning laser ophthalmoscopy images in a mouse retina of an animal expressing cyan fluorescent protein (CFP) under the Thy-1 promoter. Optic nerve head transection (ONT) was performed after obtaining the baseline (BL) image. Longitudinal images obtained 3, 7, 10 14 and 21 days after ONT. The numbers at the bottom right of each image after BL show the proportion of CFP+ cells surviving compared to BL in this animal.

The longitudinal *in vivo* estimates of the proportion of CFP+ cells surviving after ONT is shown in Fig. 3. All animals demonstrated a similar pattern of decay with a mean of approximately 10% of cells surviving at 21 days after ONT (Fig. 3). The decline in CFP+ cells was best fit by an exponential function (proportion of CFP+ cells = e^−0.12 x time after ONT^; *P*<0.01). This indicates that there was halving of CFP+ positive cells every approximately 5.8 days from any given time point. An illustrative example of an animal imaged serially up to 21 days after ONT is shown in Fig. 4.

**Figure 5:**
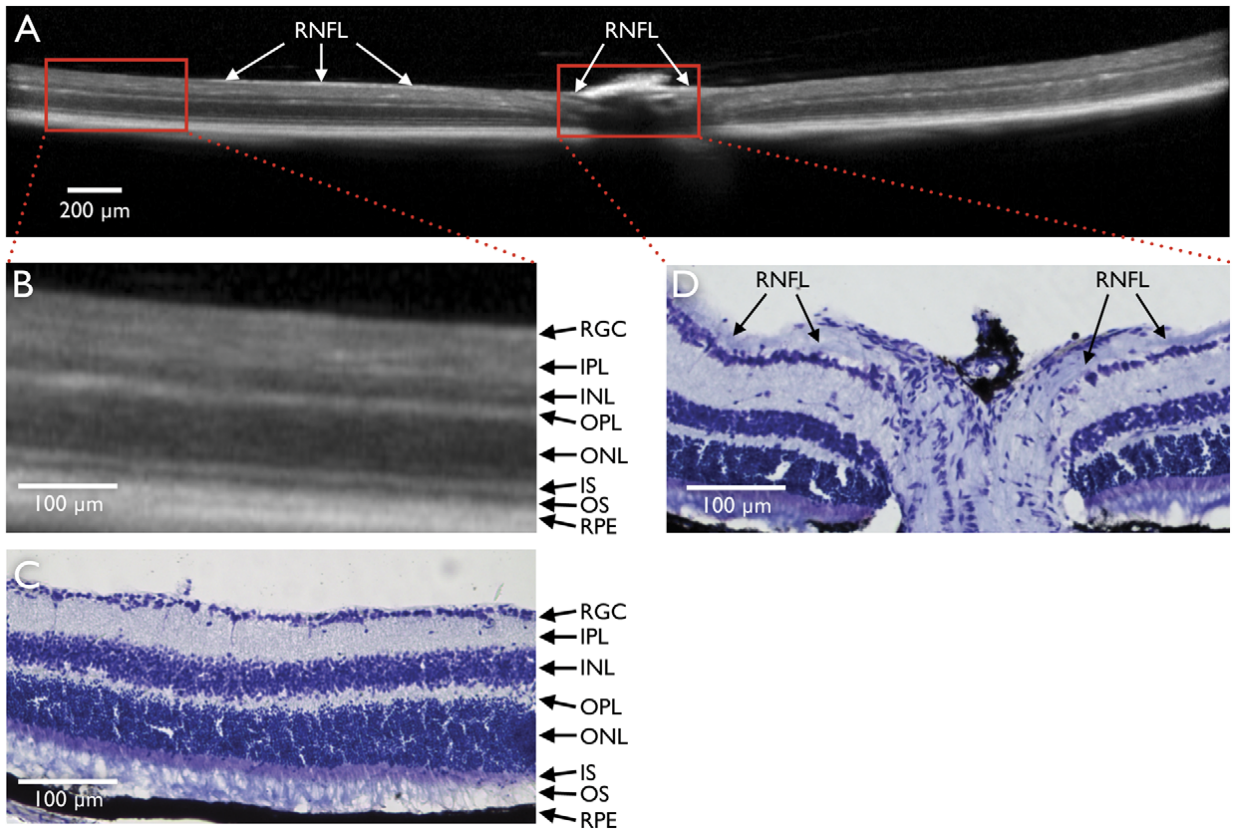
Spectral domain optic coherence tomography (SD-OCT) and corresponding histological section. SD-OCT B-scan centred on the optic nerve head obtained at baseline (A). Magnified portions of B-scan approximately 1.9 mm from the centre of the optic nerve head (B) and corresponding histological section (C) at the same location. Layers of the retina are clearly visible in the SD-OCT scan, however, at this distance from the optic nerve head, the retinal nerve fibre layer (RNFL) is not consistently visible with either SD-OCT or histology. In the optic nerve head, the SD-OCT signal is obscured by the retinal vessels, At some locations in the SD-OCT scan (A) and in the histological section (D) close to the optic nerve head, the RNFL (arrows) is visible. RGC, retinal ganglion cell layer; IPL, inner plexiform layer; INL, inner nuclear layer; OPL, outer plexiform layer; ONL, outer nuclear layer; IS, inner segments of photoreceptors; OS, outer segments of photoreceptors; RPE, retinal pigment epithelium.

**Figure 6:**
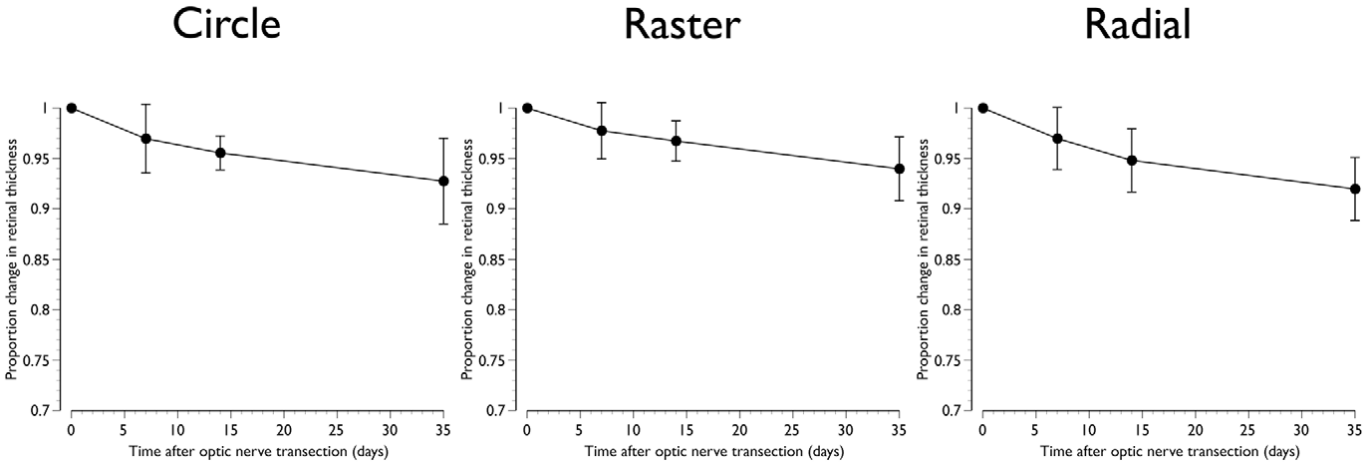
Proportional change in retinal thickness after optic nerve transection. Mean data obtained from the circle, raster and radial scanning patterns. Error bars represent standard deviations.

**Figure 7:**
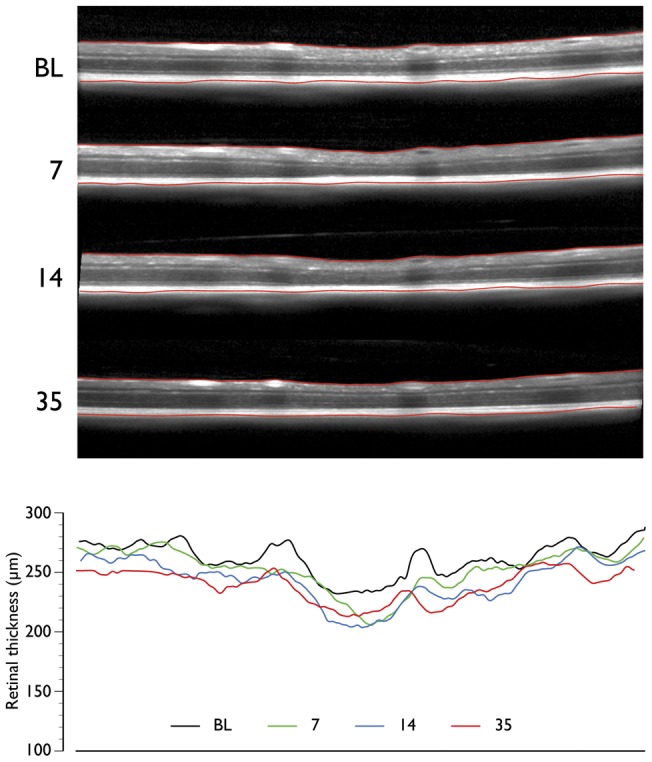
Longitudinal spectral domain optical coherence tomography (SD-OCT) scans. SD-OCT B-scans at the same line in a raster scan at baseline (BL) and 7, 14 and 35 days after optic nerve head transection (top panel). The segmentation of the retina is also shown (red lines demarcating the inner limiting membrane and retinal pigment epithelium). Retinal thickness values at corresponding time points at corresponding locations (bottom panel).

A comparison between SD-OCT and retinal histology is shown in Fig. 5. SD-OCT reliably detected retinal layers identified by histology. However, because in mice, the RNFL immediately outside the optic nerve head is thin, it could not be consistently identified and segmented for quantification in the SD-OCT images (Fig. 5). The longitudinal *in vivo* changes in total retinal thickness obtained with the three patterns of SD-OCT scanning are shown in Fig. 6. There was a linear decrease in retinal thickness (*P*<0.01) with mean (SD) reductions of 7.3 (4.2)%, 6.0 (3.2)% and 8.0 (3.1)% for the circle, raster and radial scanning patterns respectively 35 days after ONT (Fig. 6). An illustrative example with retinal segmentations of an animal imaged serially after ONT with the raster scanning pattern is shown in Fig. 7.

The total variability of retinal thickness measurements, expressed as standard deviations, in control animals imaged over three time points, expressed as the standard deviation, had a mean (range) of 0.90 (0.24–1.53)% of retinal thickness. The respective within-session values were 0.31 (0.02–1.81)%. Variability measurements for the three individual scanning patterns are shown in Table 2.

**Table 2:** Variability of retinal thickness measurements with SD-OCT*.

	Total variability	Within-session variability
Pattern 1: Circle	0.85 (0.24, 1.53)	0.11 (0.02, 0.31)
Pattern 2: Raster	0.91 (0.58, 1.35)	0.55 (0.05, 1.81)
Pattern 3: Radial	0.93 (0.60, 1.48)	0.24 (0.06, 0.046)

* values shown are mean (minimum, maximum) standard deviation as a percentage of the mean retinal thickness.

**Figure 8:**
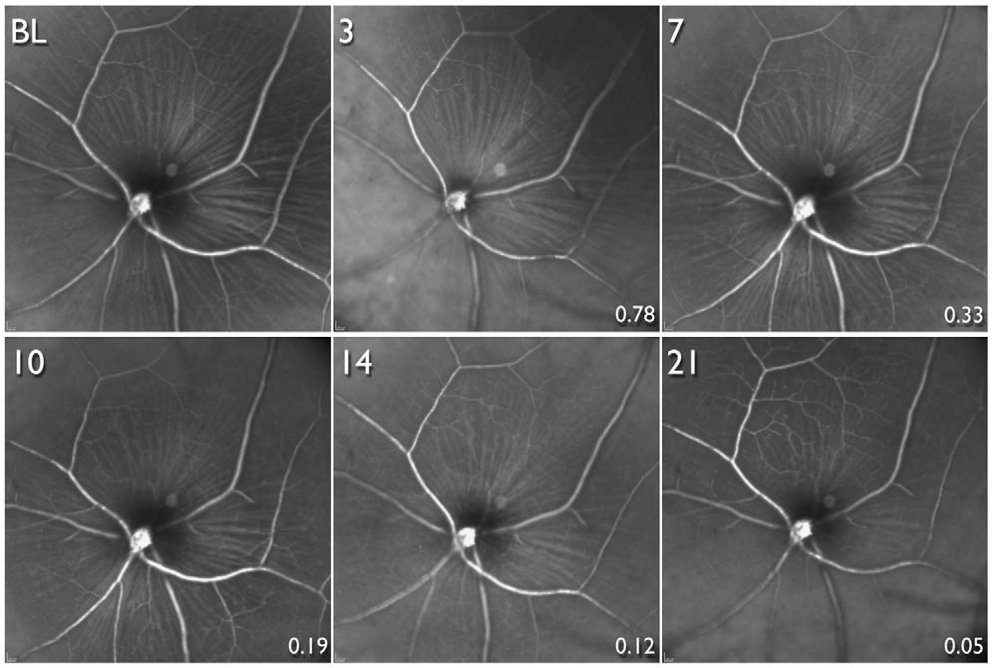
Longitudinal infrared scanning laser ophthalmoscopy images. Images are from the same animal, and at the same time-points and location (but focused on the retinal nerve fibre layer, RNFL) as the animal shown in Fig. 4. Optic nerve head transection was performed after obtaining the baseline (BL) image. Longitudinal images obtained 3, 7, 10 14 and 21 days after optic nerve transection. The RNFL appears unaltered until there was substantial loss of cyan fluorescent protein positive (CFP+) cells. The numbers at the bottom right of each image after BL show the proportion of CFP+ cells surviving compared to BL in this animal.

Longitudinal infrared images corresponding to the same time points after ONT in the same animal in Fig. 4 are shown in Fig. 8. The RNFL is clearly visible in the baseline image and up to 10 days after ONT, a time point when only 19% of CFP+ cells in this animal (and a mean of 30% across all animals) survived. However, only at 14 and 21 days after ONT was the RNFL considerably attenuated with the retinal vasculature becoming more prominent.

## Discussion

This study has demonstrated the potential of imaging techniques to longitudinally monitor changes in the retina after acute optic nerve injury in mice. Specifically, the techniques described allow quantification of cells expressing CFP under the control of the Thy1 promoter by CSLO, quantification of retinal thickness by SD-OCT and visualization of the RNFL by CSLO. Mounting the CSLO/SD-OCT camera in a stereotaxic frame, thus allowing accurate alignment between the axis of rotation of its objective and the centre of the mouse pupil, ensured that off-axis optical aberrations were minimized and that the maximum amount of laser light entered the pupil.

The results show that after ONT, there was an exponential decline in CFP+ cells with an approximately 50% progressive loss after 5.8 days from any time point. This pattern of loss corresponds approximately to the survival of RGCs after ONT in both rat [14], [15] and mouse [16], [17] obtained from cross-sectional counts of RGCs labeled retrogradely with a fluorescent tracer. With the same strain of transgenic mouse used in the present study, Leung and colleagues studied the time-course of loss of CFP+ cells after optic nerve crush in animals followed for up to 50 weeks. [18] They demonstrated that there was a rapid decline with 19% of CFP+ cells surviving after 7 days and 11% at 14 days after injury. At these time points, the respective figures from the present study were 45% and 20% (Fig. 3). Hence, interestingly, a complete transection of the optic nerve demonstrated a higher number of surviving CFP+ cells compared to a relatively less traumatic optic nerve crush injury. These differences could have at least partially arisen as a result of the different imaging techniques; specifically filter specifications for excitation and detection, and instrument type, in the two studies. An alternative explanation could be related to the specificity of CFP+ labeling to RGCs. While Thy1 is expressed primarily by RGCs, [8], [9] other cells, principally amacrine cells, [19] also express it. Furthermore, Thy1 expression in RGCs changes after injury, [20]–[22] however, the degree of change may depend on the type of injury and therefore confound the *in vivo* quantification of RGCs. We have shown that CFP is taken up by phagocytic microglia after ONT [9] which may also impact the specificity of CFP+ cells to RGCs. In ischemia-reperfusion injury of the retina (intraocular pressure elevation of 115 mm Hg for 90 mins), Leung and colleagues showed that there was no significant progressive loss of CFP+ cells after the initial loss at 7 days after injury. [23] Hence the longitudinal profile of loss of CFP+ cells depends on the type of injury.

We demonstrated a modest but significant reduction in overall retinal thickness of 6–8%, depending on the scanning protocol, at 35 days after ONT with SD-OCT. The mean total variability of retinal thickness measurements was less than 1%, and the within-session variability was even lower. Hence, the observed thickness changes are a result of ONT. Since ONT results in loss of RGCs and their axons, damage likely restricted to the RNFL and RGC layer. Because of the thin RNFL in the mouse eye (Fig. 5) we were unable to successfully segment the RNFL and RGC layers in the SD-OCT images, hence the origin of the modest reduction in retinal thickness is unclear. Histological evaluation of the retinal layers after ONT demonstrates negligible or no change in layers external to the RGC and RNFL layer, [24] hence it is possible that changes we demonstrated in total retinal thickness also originate in these layers, however, because RGC synapses are located in the inner plexiform layer, changes in this layer cannot be ruled out. However, since histological studies are cross-sectional in nature with comparisons with the fellow eye as control, non-detectable longitudinal changes in other retinal layers cannot be ruled out. We did not evaluate the correlation between SD-OCT measured and histologically measured changes in retinal thickness. Our objective was to study the time-course of changes in the same animal after ONT, which is not possible with histological measures. Performing such correlations would require a time-course to be constructed with multiple animals, leading to potential errors. Furthermore, there are additional potentially significant errors in co-localizing the same retinal section as the SD-OCT line scan, and rotation of the globe and tissue shrinkage after fixation making such correlations problematic. Finally in order to maintain good animal condition by minimizing image acquisition time, we did not perform CSLO and SD-OCT in the same animal. We did not compare the longitudinal change in CFP+ cells and retinal thickness and therefore our results are not compromised by the experimental design.

Previous investigators have evaluated mouse retinal thickness with SD-OCT. [25], [26] Other studies have examined the effects of experimental damage on retinal thickness in rodents, however, the models used varied. [27], [28] In an ocular hypertensive model in rat, Guo et al. [28] showed a 9% reduction in overall retinal thickness at 8 weeks after induction of ocular hypertension. However, the most profound reduction occurred in the outer nuclear layer, [28] contrary to findings from other studies indicating that damage with this model is limited to the RGC layer. [7], [29], [30] Gabriele et al. [27] showed an initial increase in retinal thickness following optic nerve crush in mouse, followed by a subsequent reduction but the mean change at 35 days was less than 3% compared to baseline. Because the segmentation procedure used by Gabriele et al. [27] was not illustrated with representative B-scan images, the results from this study are not easily comparable to the present one.

Recent research demonstrates that RGCs die in a compartmentalized manner such that mechanisms responsible for death of the cell body and dendrites may be different from those responsible for axonal loss. [31], [32] The compartmentalized loss of RGCs may vary according to the mechanism of damage, for example, ocular hypertension, ONT and ischemia-reperfusion. Consequently, the appearance of the RNFL may depend on the mechanism of damage. The visibility of the RNFL [33], [34] and reduction in thickness [35], [36] is thought to be one of the earliest clinical manifestations of glaucoma. Surprisingly, in our study, the appearance of the mouse RNFL appeared unchanged until there was substantial (∼70% or more) RGC loss (Fig. 8). These findings could be due to the acute nature of ONT. While RGCs die soon after ONT, there may be relative little change in the structural appearance of the RGC axons, even though they may be functionally compromised. Further research with other models of both acute and chronic RGC damage is necessary to determine whether RNFL appearance is altered with lesser RGC loss.

*In vivo* imaging of the mouse retina provides a valuable tool for studying a variety of structural alterations following experimental damage or for evaluating the benefits of neuroprotective strategies. Additional research is necessary to characterize and distinguish the effects on these structural measurements as a result of the various experimental methods of causing RGC loss.
